# Phosphonates enantiomers receiving with fungal enzymatic systems

**DOI:** 10.1186/s12934-021-01573-8

**Published:** 2021-04-07

**Authors:** Monika Serafin-Lewańczuk, Małgorzata Brzezińska-Rodak, Katarzyna Lubiak-Kozłowska, Paulina Majewska, Magdalena Klimek-Ochab, Tomasz K. Olszewski, Ewa Żymańczyk-Duda

**Affiliations:** 1 Department of Biochemistry, Molecular Biology and Biotechnology, Laboratory of Biotechnology, Wrocław University of Science and Technology, Wrocław, Poland; 2 Department of Physical and Quantum Chemistry, Wrocław University of Science and Technology, Wrocław, Poland

**Keywords:** Biotransformation, Heterocyclic phosphonates, Fungi, Yeast

## Abstract

**Background:**

Phosphonates derivatives are in the area of interests because of their unique chemical-physical features. These compounds manifest variety of biological interactions within the sensitive living cells, including impact on particular enzymes activities. Biological “cause and effect” interactions are based upon the specific matching between the structures and/or compounds and this is usually the result of proper optical configurations of particular chiral moieties. Presented research is targeted to the phosphonates with the heteroatom incorporated in their side functionalities. Such molecules are described as possible substrates of bioconversion for the first time lately and this field is not fully explored.

**Results:**

Presented research is targeted to the synthesis of pure hetero-phosphonates enantiomers. The catalytic activity of yeasts and moulds were tested towards two substrates: the thienyl and imidazole phosphonates to resolve their racemic mixtures. Biotransformations conditions differed depending on the outcome, what included changing of following parameters: type of cultivation media, bioprocess duration (24–72 h), additional biocatalyst pre-treatment (24–48 h starvation step triggering the secondary metabolism). (*S*)-1-amino-1-(3-thienyl)methylphosphonate was produced with the assistance of *R. mucilaginosa* or *A. niger* (*e.e*. up to 98% and yield up to 100%), starting from the 3 mM of substrate racemic mixture. Bioconversion of racemic mixture of 3 mM of (1-amino-1-(4-imidazole)methylphosphonic acid) resulted in the synthesis of *S*-isomer (up to 95% of *e.e.*; 100% of yield) with assistance of *R. mucilaginosa.* 24 h biotransformation was conducted with biomass preincubated under 48-hour starvation conditions. Such stereoselective resolution of the racemic mixtures of substrates undergoes under kinetic control with the conversion of one from the enantiomers.

**Conclusions:**

Composition of the culturing media and pre-incubation in conditions of nutrient deficiency were significant factors influencing the results of kinetic resolution of racemic mixtures of phosphonic substrates and influencing the economic side of the biocatalysis e.g. by determining the duration of whole biocatalytic process.

**Graphical abstract:**

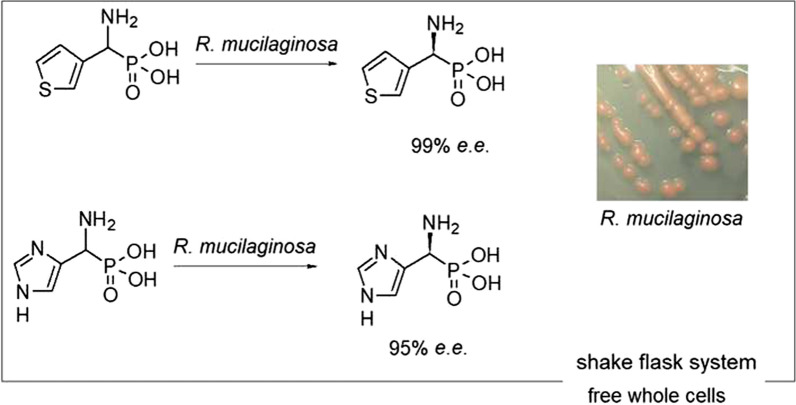

## Background

Asymmetric organic synthesis is one of the most demanding challenges of modern organic chemistry due to the fact that the majority of chiral compounds with biological activity, show the desired effect only in optically pure form. Available chemical methods often require the use of harmful reagents or expensive asymmetric catalysts [1], hence the biocatalytical methods are currently of great interest. Such approach can be simpler than the chemical one and is often one-step process. The use of biocatalysis for the synthesis of chemical compounds of biological importance in a stereoselective manner is an attractive alternative, comparing to traditional chemical methods [2, 3]. Among all microorganisms, lower fungi are attractive group of eukaryotic organisms, differing in both physiological and morphological qualities, so their enzymatic systems are of great biocatalytic potential. These microorganisms have relatively low nutritional requirements and high adaptability for various environments, what significantly increases the spectrum of their applications. Their high adaptability is related to the possibility of quick correction of the main metabolic pathways, to the activation of alternative pathways or to the production of secondary metabolites. Due to such metabolic “flexibility”, these organisms are able to utilize various sources of carbon and/or energy or nitrogen, and thus they can also transform structurally different xenobiotics. A well-thought-out and rationally planned method of biocatalyst cultivation or its subsequent modification as well as the reaction medium modification allow changing the activity of the fungal enzymatic systems and matching them to specific substrates.

The spatial arrangement of substituents connected with a chiral carbon atom is responsible for the properties of a given derivative, especially in the area of biological activity. Among others, phosphonates are a group of compounds with diverse biological activities. They can serve as synthons for further synthesis or as active components of drugs of antibacterial, hypotensive or anti-osteoporotic features [4]. Among them, aminophosphonates, as structural analogues of natural amino acids, inhibit the activity of various enzymes, especially proteases such as HIV protease, aminopeptidase or human collagenase [5]. Synthesis of aminophosphonates and their derivatives is quite common and well documented [6–12] but obtaining the heterophosphonates, especially as optically pure isomers is still a current research topic. Aminophosphonic acids containing heterocyclic functionalities in their structure can be used as building blocks applied for the synthesis of biologically active compounds, mainly pharmaceuticals. The presence of a heterocycle decreases toxicity of this structures compared to the aromatic counterparts, widening their applicability for medical chemistry [13].

**Fig. 1:**
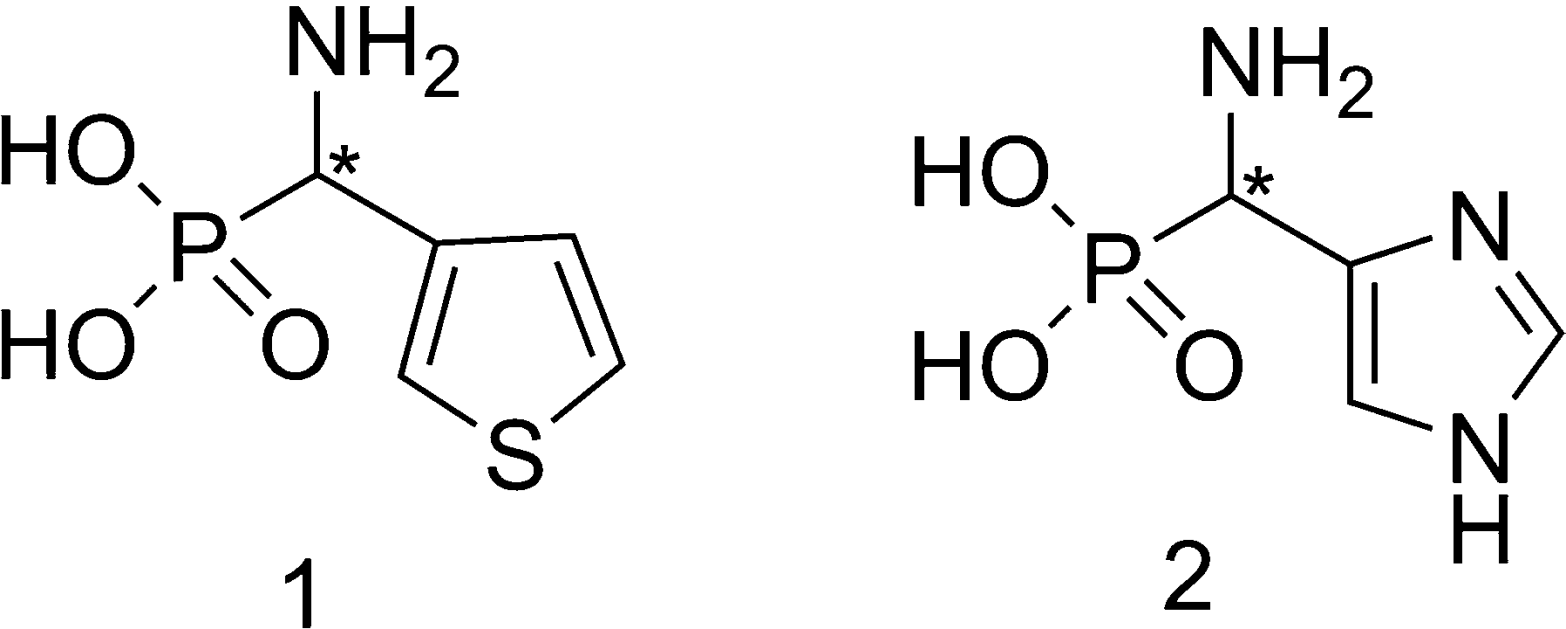
Substrates: 1-amino-1-(3-thienyl)methylphosphonic acid (**1**) and 1-amino-1-(4-imidazole)methylphosphonic acid (**2**)

Thus, the aim of this study was to synthesize the optically pure isomers of heterophosphonates *via* the biocatalyzed resolutions of the racemic mixtures of the following substrates: 1-amino-1-(3-thienyl)methylphosphonic acid (**1**) and 1-amino-1-(4-imidazole)methylphosphonic acid (**2**) (Fig. 1). Applied fungal enzymatic systems carried out the oxidative deamination of one from the enantiomers remaining the other one unreacted. Optically pure products are valuable building blocks in the synthesis of biologically active compounds such as pharmaceuticals or pesticides. They serve as chiral motives in such structures.

## Results

### Alamar Blue assay

Alamar Blue Assay is widely used in studies on cells viability and cytotoxicity of xenobiotics. Alamar Blue method is based upon the measurement of the ability of biological system to reduce particular substrate—it has been used to assess the viability of the bacteria, yeasts and fungal cells also human and animal cell lines and is an integral part of drug discovery process. Living cells enzymes reduce blue resazurin from Alamar Blue to pink resorufin [14].

Alamar Blue Assay was applied as antimicrobial susceptibility test to measure biological activity of tested heterophosphonates to check the range of tolerance of particular organisms towards these compounds. Experiments were performed on selected model pro- and eukaryotic microorganisms, i.e. representatives of mold fungi, yeasts, bacteria and cyanobacteria.

Solution of compound **1** and **2** were prepared with cultivation medium in wide range of concentrations (2–10000 µg/mL).

**Table 1:** Antimicrobial activity of 1-amino-1-(3-thienyl)methylphosphonic acid (**1**) and 1-amino-1-(4-imidazole)methylphosphonic acid (**2**)

Microorganism	MIC [µg/mL] for compound **1**	MIC [µg/mL] for compound **2**
*B. subtilis*	8000	1250
*E. coli*	5000	1250
*S. bigranulatus*	5000	512
*R. mucilaginosa*	2000	1024
*A. niger*	10,000	5000

Despite having active groups, such as imidazole and tiophene residues, the tested compounds showed no inhibitory activity in the tested range of concentrations. In most cases the minimal inhibitory concentration (MIC) was above 1024 µg/mL (Table 1). Only cyanobacteria *S. bigranulatus* was sensitive to applied chemicals, from all tested strains—the effective inhibitory concentration of 1-amino-1-(4-imidazole)methylphosphonic acid (**2**) was 512 µg/mL. These results confirmed the catalytic applicability of tested organisms towards phosphonates derivatives.

### Biotransformation

Based both on results described above and previous experience in the field of phosphonate biocatalysis fungal biocatalysts were selected and applied for the resolution of racemic mixtures of following phosphonates derivatives: 1-amino-1-(3-thienyl)methylphosphonic acid (**1**) and 1-amino-1-(4-imidazole)methylphosphonic acid (**2**).

### Biotransformation of the racemic 1-amino-1-(3-thienyl)methylphosphonic acid (**1**)

12 representatives of filamentous fungi and yeasts (*R. mucilaginosa, F. oxysporum, T. variabilis, B. bassiana*, *C. elegans*, *R. oryzae*, *P. commune*, *A. niger*, *P. funiculosum A. parasiticus, M. circinelloides* and *P. citrinum*) were screened towards phosphonic substrates. Five of tested strains were active towards 1-amino-1-(3-thienyl)methylphosphonic acid (**1**) (Tables 2 and 3). Different process conditions were applied to improve preliminary results. Following parameters were variable: duration of the biotransformation, type of the cultivation media and the number of the biocatalysis stages with e.g. the introduction of additional preincubation period conducted under starvation conditions. The most effective approaches were as follows: yeasts biocatalyst *R. mucilaginosa* cultivated for 5 days on PDB/PDB2 medium and preincubated under starvation conditions for 24/48 hours was employed for 24/48 hours biotransformations and product of (*S*)-configuration was received with the enantiomeric excess up to 99% with conversion degree of 50% (Table 3). Whereas, enantiomeric excess over 89% was obtained in process with *A. niger* conducted after preincubation of fungal mycelium under starvation conditions for 24 h and biotransformation was completed within 72 h (Table 2).

**Table 2:** Results of the biotransformation of 1-amino-1-(3-thienyl)methylphosphonic acid (**1**)

Biocatalyst	Cultivation medium	Time of cultivation (h)	Time of preincubation under starvation conditions (h)	Time of biotransformation (h)	*e.e.* ^a,b^ [%]
*R. mucilaginosa*	PDB	72	0	72	35
		72	24	72	28
	YM	72	0	72	33
*A. niger*	PDB	96	0	24	31
		96	24	24	52
		96	0	72	27
		**96**	**24**	**72**	**89**
	MEP	96	0	72	6
*F. oxysporum*	PDB	96	0	72	14
		96	24	72	35
	MEP	96	0	72	42
		96	24	72	30
*M. circinelloides*	PDB	96	0	72	31
		96	24	72	49
*A. parasiticus*	PDB	96	0	72	< 5
		96	24	72	12

Bold values indicate best result

^a^Racemic mixtures were enriched in (*S*)-enantiomer. ^b^The yield of bioconversion is adequate to the evaluated *e.e.* and in case of pure enantiomer synthesis the yield is 100%

**Fig. 2:**
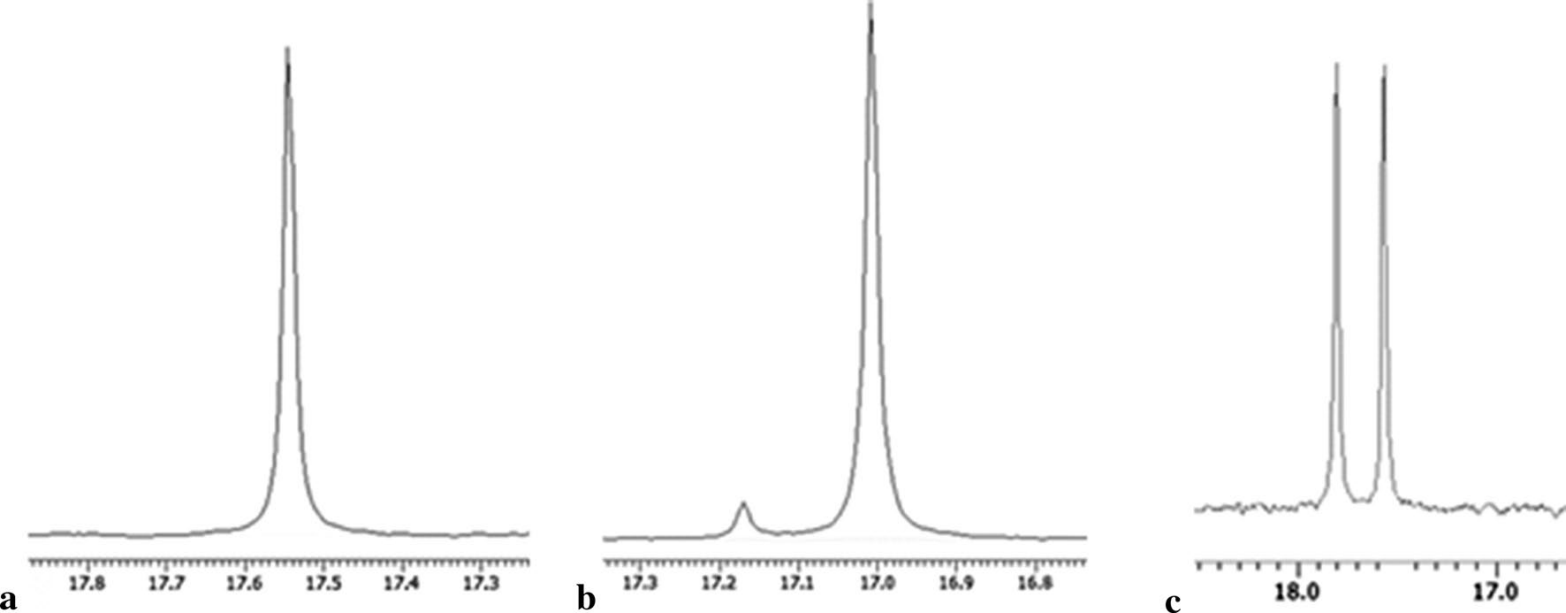
^31^PNMR spectra recorded with the addition of α-CD used as chiral solvating agent at pD ≈ 11–12; after biotransformation of compound **1** catalyzed by **a** *Rhodotorula mucilaginosa* (99% *e.e.*) **b** *Aspergillus niger* (89% *e.e*.); **c** ^31^P NMR spectrum of starting racemic mixture of compound **1** recorded with addition of α-CD at pD ≈ 12

Recorded results confirmed that following factors: applied culturing medium, time of cultivation or preincubation step introduction significantly influence the bioconversion efficacy. Application of biomass of *R. mucilaginosa* (after 5-day of cultivation followed by 24 h preincubation under deficiency of nutrients) allowed increasing the enantioselectivity of the process from 35 to 99% of *e.e.* (Table 3). In every case, the enantiomeric excess was evaluated based upon the ^31^P NMR spectroscopy (Fig. 2).

**Table 3:** Results of the biotransformation of 1-amino-1-(3-thienyl)methylphosphonic acid (**1**) with *Rhodotorula mucilaginosa*

Biocatalyst	Cultivation medium	Time of cultivation [h]	Time of preincubation under starvation conditions [h]	Time of biotransformation [h]	*e.e.* ^a,b^ [%]
*R. mucilaginosa*	PDB	72	0	72	35
		72	24	72	28
		120	0	24	94
		**120**	**24**	**24**	**96**
		120	48	24	78
	PDB2	120	0	24	39
		**120**	**48**	**24**	**≥ 98**
		**120**	**48**	**48**	**≥ 99**
	YM	72	0	72	33

Bold values indicate best result

^a^Racemic mixtures were enriched in (*S*)-enantiomer. ^b^The yield of bioconversion is adequate to the evaluated *e.e.* and in case of pure enantiomer synthesis the yield is 100%

### Biotransformation of racemic mixture of 1-amino-1-(4-imidazole)methylphosphonic acid (**2**)

**Fig. 3:**
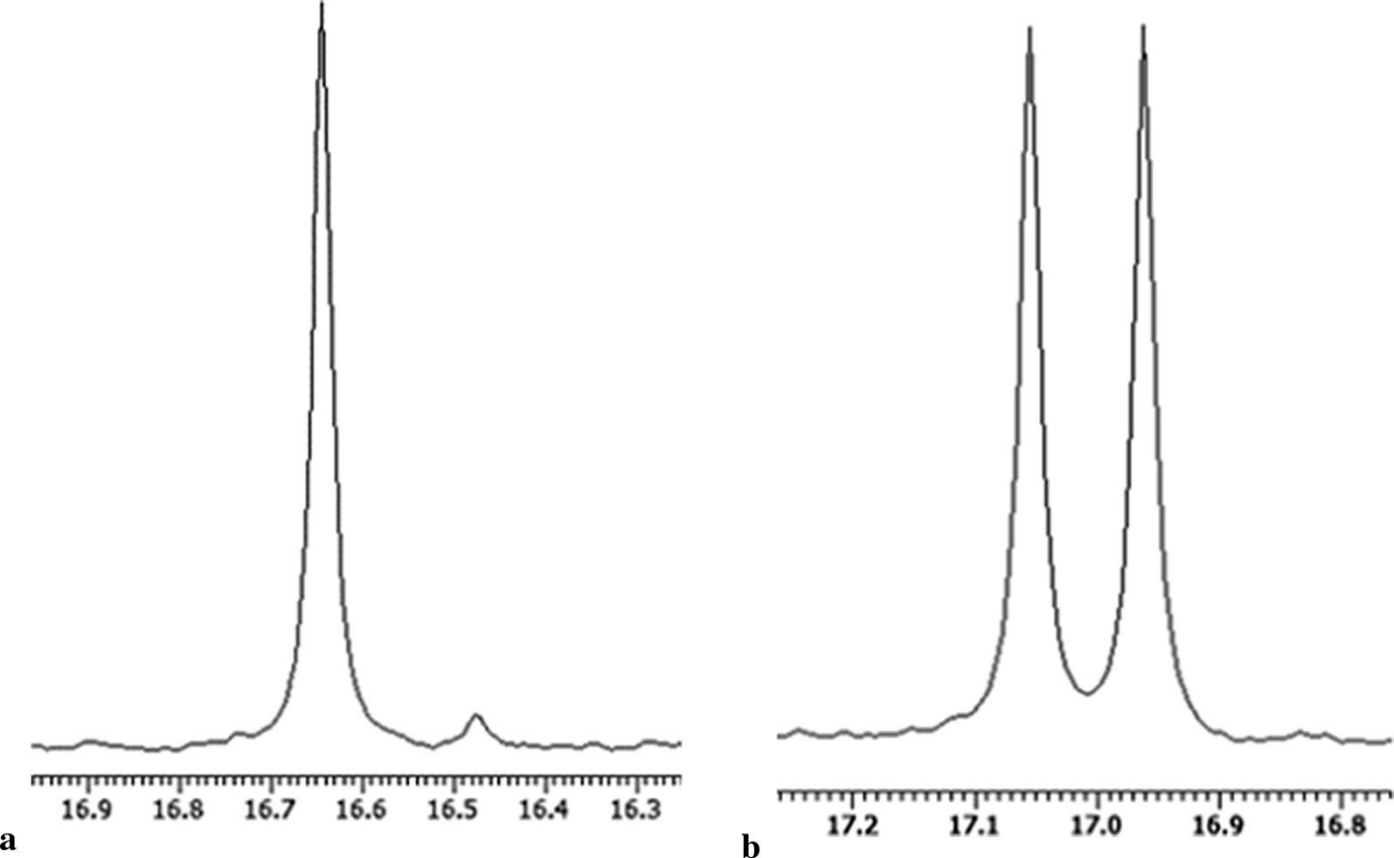
**a** ^31^PNMR spectra recorded with the addition of α-CD as chiral solvating agent at pD ≈ 11–12; **a** after biotransformation of compound **2** catalyzed by *Rhodotorula mucilaginosa* (95% *e.e*.); **b** starting racemic mixture of compound **2**

Biotransformation of 1-amino-1-(4-imidazole)methylphosphonic acid (**2**) was effective only with the application of *R. mucilaginosa* cells (Table 4). Yeasts were employed as biocatalysts after 5-days cultivation and then 2-days of preincubation under starvation conditions. These resulted in the isomers mixture enrichment to 95% of *e.e*. by enantiomer (*S*) (Fig. 3).

**Table 4:** Results of the biotransformation of 1-amino-1-(4-imidazole)methylphosphonic acid (**2**) by *Rhodotorula mucilaginosa*

Biocatalyst	Cultivation medium	Time of cultivation [h]	Time of preincubation under starvation conditions [h]	Time of biotransformation [h]	*e.e* ^a,b^ [%].
*R. mucilaginosa*	PDB	72	0	72	32
		72	24	72	24
		72	24/0	24	25
		72	24	24	9
		120	0	24	92
		120	24	24	82
		**120**	**48**	**24**	**95**
		120	48	48	75
	PDB2	**120**	**48**	**24**	**93**
		120	48	48	79
	YM	72	0	72	33

Bold values indicate best result

^a^Racemic mixtures were enriched in (*S*)-enantiomer. ^b^The yield of bioconversion is adequate to the evaluated *e.e.* and in case of pure enantiomer synthesis the yield is 100%

## Configurational assignments

The determination of the absolute configuration of 1-amino-1-(3-thienyl)methylphosphonic acid (**1**) was performed according to Mosher’s method [15]. Investigation was based upon the double derivatization. Analysis started with compound **1** partially transformed by *R. mucilaginosa.* Introduced spectroscopic method allowed to recognize products as non-equimolar mixture of enantiomers [(*S*):(*R*)] of compound **1** of molar ratio 3.5:1.0. Thus, after separation of the bioconversion products by MPLC system, compound **1** was acylated by (*S*)-(-)-MTPA resulting in a mixture of (*S, S*): (*R,S*) isomers of Mosher’s amide **3** with molar ratio of (2.1:1.0).

**Fig. 4:**
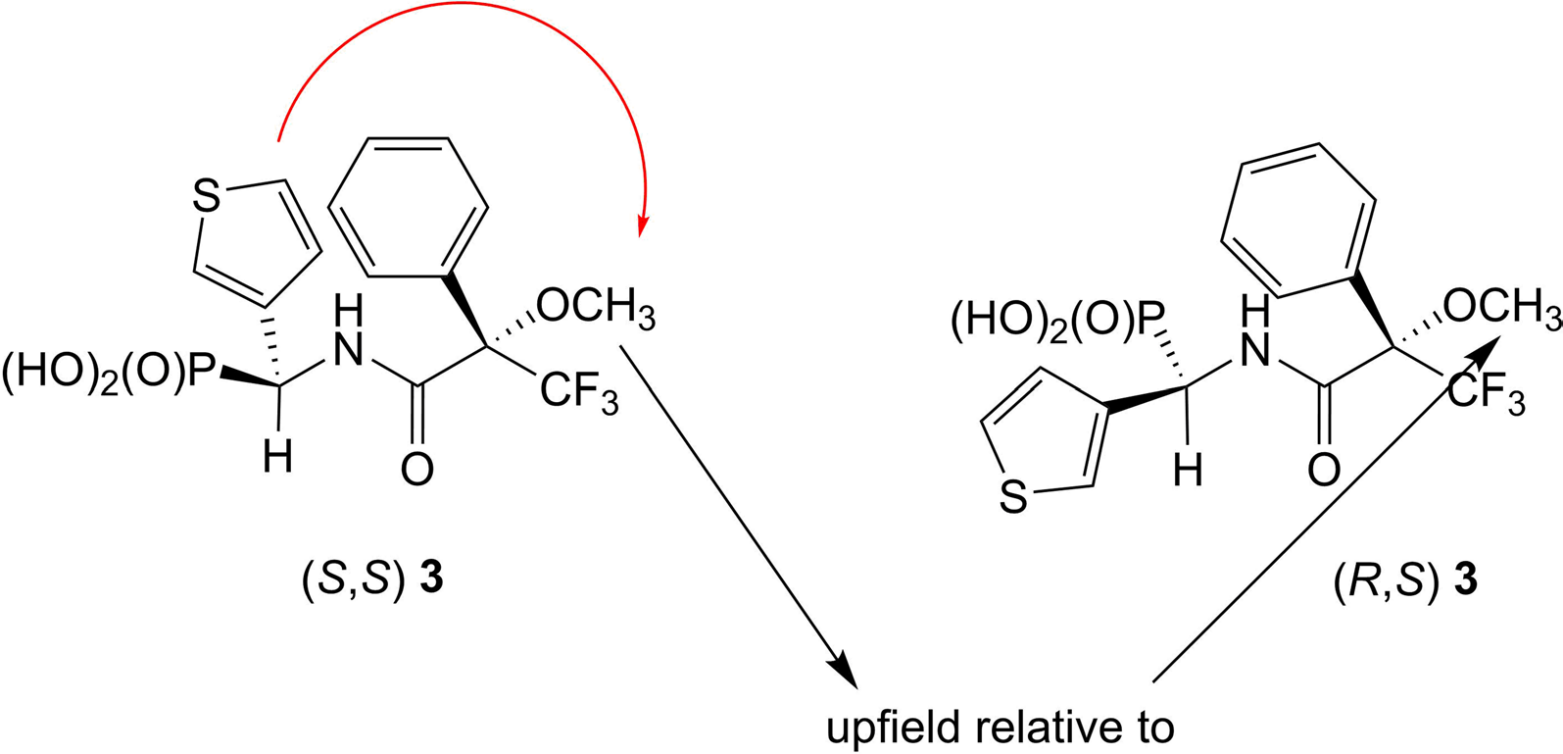
Anisotropic effect of thienyl group on hydrogen atoms forming the methoxy group

**Fig. 5:**
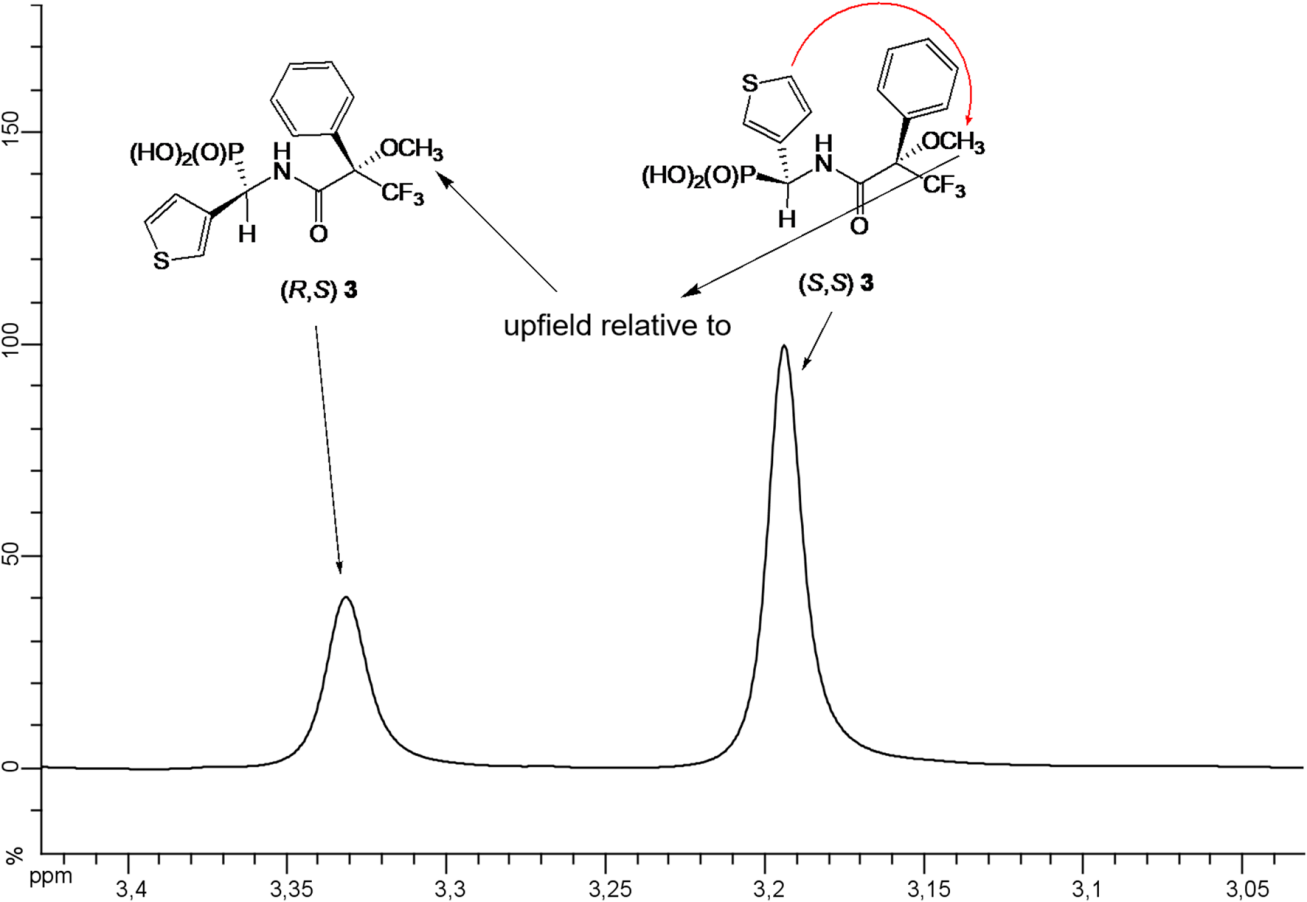
^1^H NMR of amide **3** (anisotropic effect of thienyl group on hydrogen atoms form methoxy group)

^1^H NMR chemical shifts of methoxy group (OC**H**_3_) of compound **3** were assigned as follows: (*R,S*)—3.33 ppm; (*S,S*)—3.19 ppm. As it can be seen the signals becoming from hydrogen atoms of methoxy group of isomer of (*S*)-configuration at α-carbon atom are upfield if compared to (*R*)-isomer (Figs. 4 and 5).

**Fig. 6:**
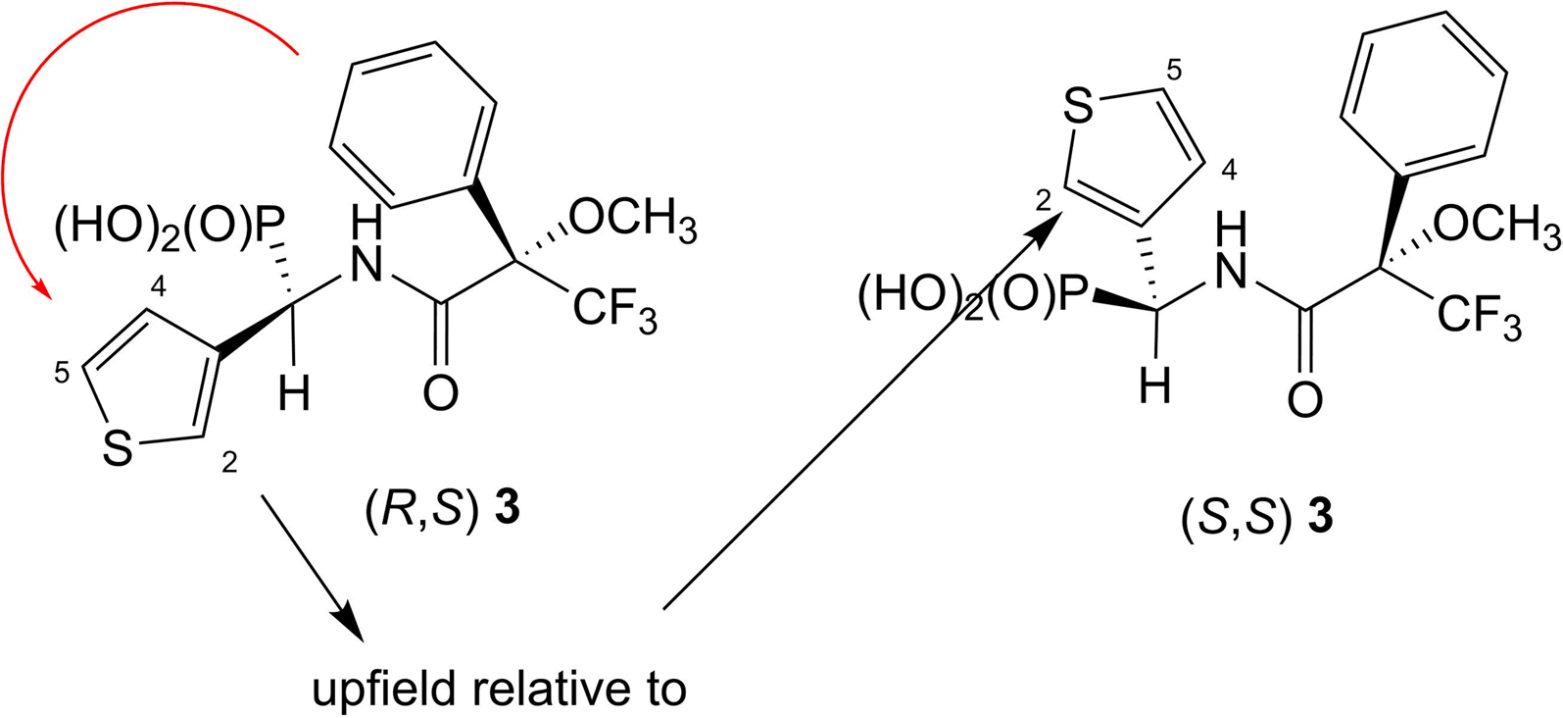
Anisotropic effect of phenyl group on hydrogen atoms of thienyl group

**Fig. 7:**
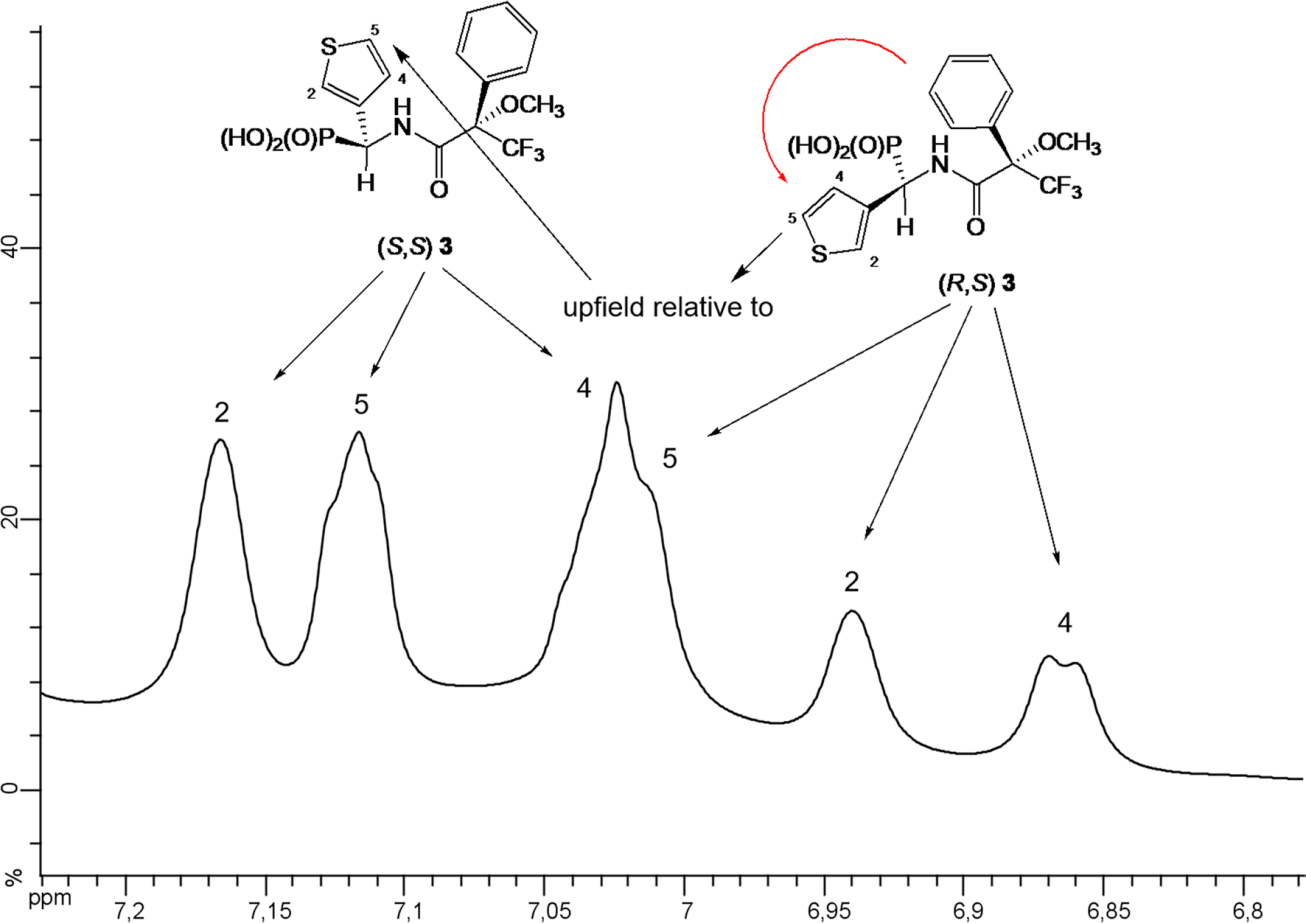
^1^H NMR of amide **3** (anisotropic effect of phenyl group on hydrogen atoms of thienyl group)

Furthermore, ^1^H NMR chemical shifts of thienyl group of compound **3** were assigned as follows: (*S,S*)—7.02 ppm (H-4), 7.12 ppm (H-5), 7.17 ppm (H-2); (*R,S*)—6.86 ppm (H-4), 6.94 ppm (H-2), 7.01 ppm (H-5). In this case it can be seen that the signals derived from hydrogen atoms of thienyl group of isomer of (*R*)-configuration at α-carbon atom are upfield if compared to (*S*)-isomer (Figs. 6 and 7).

Comparison of all the spectra allowed to assign the absolute configuration of enantiomers of compounds **1**.

## Discussion

Chiral phosphonates are a group of compounds with such a wide potential of applications that synthesis of their enantiomers in optically pure form is being sought as well as with chemical as biocatalytical methods. Employing biological systems is competitive to other approaches and can be effective regarding the enzymes features. Desired and applicable biological activity of phosphonates is predominantly associated with the inhibition of the activity of the particular enzymes e.g. proteases [5]. This is a reason, that finding the right biocatalyst capable of transforming these compounds is not a simple matter. Therefore, usually for each substrate, the type of biocatalyst and the conditions for both culturing of microorganism and conducting the biotransformation process itself, should be selected individually. Lower fungi have been selected for phosphonate transformation due to the simplicity of their cultivation, biomass productivity, enormous variability and activity of the fungal enzymatic systems. These last ones are susceptible to manipulation by environmental physical-chemical factors. Also, fungal metabolic pathways can be easily redirect to secondary ones, what is associated with the activation/production of enzymes of very specific activities. This facilitates and sometimes makes possible the bioconversion of non-physiological compounds. The settings of the parameters of biotransformation of substrate **1**, started from culturing of 5 fungal strains under different conditions such as media or cultivation duration or preincubation step (Table 2). Screening experiments pointed *A. niger* as biocatalysts assuring the satisfactory result for conversion of substrate **1** (Table 2). Product was received with the enantiomeric excess of 89% *e.e*. (following conditions were applied: 96 h of growing on PDB medium, 24 h of pre-incubation under starvation condition and 72 h of transformation). PDB is one of the most commonly used media for the isolation and cultivation of fungi providing effective biomass formation and it is characterized by high carbon : nutrient ratio, influencing the microbial activity [16]. In the case of other microorganisms (*R. mucilaginosa, F. oxysporum, M. circinelloides, A. parasiticus*) and regardless of the application of an additional starvation step, the final effect was rather poor: the enantiomeric enrichment of the isomers mixtures ranged between 5 and 49% of *e.e.* Introduced starvation period forced the cells to use every available source of biogenic elements for cell purposes, also nutrition deficiency impacts on the metabolism of the fungi switching on some rescue enzymes of unusual activities and substrates specificities [17]. These are the reason of observed conversion of the xenobiotic substrates under such unfavorable for viable cells conditions as e.g. water solution of phosphonic substrates. As it is reported in the literature, fungal enzymes secretion is initiated when the fungus grows on substrates with low amino acids and sugar contents since transport-related gene families are expressed to utilize external nutrient resources for its survival [18]. To assimilate the only source of energy *Aspergillus* sp. switched on its redox activity [19] and started the mineralization of one from the isomers of accessible organic compound *via* enantioselective oxidative deamination of (*R*) hetero-phosphonate [20], what proceeds to mineralization of one of the enantiomers of the substrate and the second one remains unreacted. This path is confirmed by the ^31^P NMR spectra (Fig. 2b), where the only recorded signals become from the non-converted substrate enantiomer (*S*). The next efforts were directed to achieve better enantiomeric excess—above 90% and more economic procedure e.g. shorter time of the whole experiment. Further experiments confirmed that results described above are the best possible ones for *A. niger*. Considering previous research and based upon practical experiences with other catalytic microbes, *R. mucilaginosa* was subjected to the next work. Despite the results obtained at the initial stage of the research (35% *e.e.)*, biotransformation of 1-amino-1-(3-thienyl)methylphosphonic acid (**1**) was continued with *R. mucilaginosa* because of its enormous activity noted towards previously examined phosphonate derivative: 1-amino-1-(3′-pyridyl)methylphosphonic acid [20]. In the mentioned studies, just after 24 h of biotransformation with biocatalyst pre-treated for 24 h without any nutrient components, pure (*S*)-1-amino-1-(3′-pyridyl)methylphosphonic acid (100% of *e.e*.; with yield of 100% ) was synthesized. Current research started from the selection of the optimal culturing media for *R. mucilaginosa* to allow manifesting its enzymatic potential. Three media were applied—PDB commercially available, PDB2- hand-made according to procedure of DSMZ for medium no. 129 and special medium composed for yeast growth (YM). The cultivation on potato medium (PDB, PDB2) lasted for 5 days according to growth curve (data not shown), what allowed entering *R. mucilaginosa* culture to the late logarithmic phase of growth. Long cultivation, following the starvation period, induces the stress conditions for viable cells and, as was mentioned previously, triggers on the processes related to secondary metabolism such as: synthesis of enzymes of specific activities or activation of transport systems involved in uptaking resources from the environment. According to observations, *R. mucilaginosa* cultured on PDB medium manifests promising features as biocatalysts. Biotransformations of substrate **1** conducted directly with biomass of yeast received after growing period, resulted in a formation of the product [(*S*)-**1**] of high optical purity (94% *e.e.)*. PDB medium was also applied for parallel experiments with the introduced starvation stages, what was the pointless approach, just slightly affecting the optical purity in case of 24-hours incubation (product [(*S*)-**1**] was of 96% of *e.e*.) while 48-hours period caused partial racemization of product (**1**) (78% *e.e*. of *S*-isomer) (Table 3). This time secondary metabolism enzymes—also survival ones—were produced and were active towards both of the substrate’s enantiomers. According to known fungal defend mechanisms the longer starvation lasts the higher level of rescue enzymes is produced. Such situation resulted in observed racemization of the bioconversion products, which is possible in case of matching of enzymatic activity to xenobiotic. The lack of the basic ingredients can influence the activity of the enzymes active towards the xenobiotic in alternative way, causing the decrease or increase in the activity of the enzymes of opposite enantioselectivity, additionally the activation of other enzymes (e.g. racemases) is possible as the path of efficient substrate assimilation (both enantiomers) for cellular purposes. Experiments with *R. mucilaginosa* and substrate **1** leading to the interesting observations that applying the PDB2 medium (home-made; DSMZ recipe) requires the introduction of starvation steps to assure high conversion effectiveness (Table 3), what is in contradiction to above results, describing to the PDB case. PDB2 medium is richer in ingredients responsible for fungus growth and for the activity of the primary metabolic pathways. So, as a consequence the biomass received after culturing on such medium should be stimulated to widen its enzymatic possibilities towards the xenobiotic conversion by triggering the secondary metabolism by environmental factor such as starvation. The significance of the formula of cultivation media manifests itself in the results of substrate **1** biotransformation. Biocatalyst prepared with PDB medium and applied directly to the bioconversion process was furthermore active and selective towards phosphonic compound, then one cultured on PDB2 medium (Table 3). Kinetic resolution with biomass from PDB was highly effective towards substrate **1** (94% of *e.e*., maximal conversion degree, 24 h.), whereas biocatalyst grown on PDB2 medium under comparable conditions of biotransformation was of very poor selectivity (34% of *e.e*.). This last one was improved by the introduction of starvation step (48 h.), what resulted in the activation/synthesis of enzymes capable of efficiently resolution of the racemic mixture and obtaining product [(*S*)-**1**] of high enantiomeric excess (98–99% *e.e*—Fig. 2a; Table 3).

Resolution of the racemic mixture of substrate **2**: 1-amino-1-(4-imidazole)methylphosphonic acid was more complicated and finding the microbe capable to convert this compound was a hard matter. Among tested fungi, only *Rhodotorula* strain was active towards this substrate (**2**). The presence of two nitrogen atoms in imidazole moiety increases the electronegativity of this derivative, what can cause difficulties in transport across cells covers in filamentous fungi. *Rhodotorula mucilaginosa* as yeast differs in the cells wall chemical composition and of course in the transport systems across plasma membrane and cell wall. It seems that this is the main reason of such big difference in the activity towards imidazole derivative, between molds and yeast. Thus, the use of 3-day biomass for biotransformation, turned out to be inefficient in every mode of experiments: with/without an additional starvation stage. After accomplished of these experiments, the enantiomeric excesses in the product mixtures ranged from 9 to 32% of *e.e.* These results were crucial for understanding that enzymes involved in the substrate 2 conversion achieved the maximum of their activity later, after the culture enters into the final stage of logarithmic growth phase. The biocatalysts obtained after 5 days cultivation allowed receiving the product [(*S*)-**2**] with an optical purity above 90%. Similarly, as in the case of thiophene substrate **1** transformation, the influence of biocatalyst culture conditions on its activity towards substrate **2** was checked. Both potato media (PDB, PDB2) were tested and in this case commercially available medium proved to be slightly better. After 24 h biotransformation, carried out by 5-day culture biomass, one of the enantiomers [(*S*)-**2**] was obtained with 92% excess. The introduction of the pre-incubation stage allowed increasing the optical purity of the obtained product (48 h of starvation, 24 h of biotransformation − 95% *e.e.* Fig. 3a) (Table 4). Extending the biotransformation period led to partial racemization of the product **2** (75% of *e.e.*). This is probably a result of activation of additional enzymatic activities necessary for the assimilation of the any source of biogenic elements available in the very minimal medium (water substrate solution). Analogous experiments using biomass cultivated on PDB2 medium allowed obtaining the product [(*S*)-**2**] with 93% of *e.e.* (48 h of pre-incubation, biotransformation 24 h) and 79% of *e.e.*, respectively (48 h pre-incubation, 48 h biotransformation). Transformation of both substrates (**1** and **2**), by biomass cultivated on YM medium proved to be inefficient − 33% enantiomeric excess which proves low biocatalyst activity in relation to the tested substrates.

## Conclusions

Obtained results can be a consequence of the alteration of activity of fungal enzymatic systems in the response to the environmental factors—such as stress induced by starvation conditions or different composition of culture media (PDB, PDB2, YM). In case of *A. niger* and *R. mucilaginosa* the introduction of the starvation step before bioconversion was the crucial stage for the activity of the enzymes involved in stereoselective resolution of the racemic mixture of the phosphonic substrates as it was mentioned in detail in the discussion part of the text. Described results are the good starting point for the scaling approaches to implement biological procedures in the chiral phosphonates production.

## Methods

All chemicals were purchased from: Avantor Performance Materials Poland S.A., Sigma Aldrich, Fluka. Cultivation media were purchased from VWR Chemicals.

NMR (Nucleic Magnetic Resonance) spectra were measured on a Bruker Avance™ 600 at 600.58 MHz for ^1^H and 243.12 MHz for ^31^P or on a Jeol ECZ 400 S at 161.92 MHz for ^31^P. All analysis was carried out in D_2_O (99.9% of atom D). Chemical shifts (δ) were reported in ppm and coupling constants (*J*) were given in Hz. ^1^H NMR spectra are referenced to the central line from solvent (δ = 4.78 for water). The biotransformation products were analyzed by ^31^P NMR and ^1^H NMR.

The optical rotation was measured in 1 N NaOH using polAAr-31 polarimeter (578 nm).

MS spectra were obtained using high resolution mass spectrometer with analyzer time-of-flight (TOFMS) from LCT PremierTM XE.

### Synthesis of substrates

The aminophosphonic acids were obtained in a three-step synthesis based on addition of diethyl *H*-phosphonate to the previously prepared imine and followed by acidic hydrolysis of the resulting aminophosphonate diethyl ester.

### Synthesis of 1-amino-1-(3-thienyl)methylphosphonic acid (**1**)

Neat benzhydrylamine (1.53 mL, 8.9 mmol) was injected at room temperature to the solution of 3-thiophenecarboxaldehyde (0.78 mL, 8.9 mmol) in CH_2_Cl_2_ (15 mL) and the reaction was stirred overnight. After that time, anhydrous Na_2_SO_4_ (3.0 g) was added and the mixture was stirred for additional 0.5 h. After removal of the drying agent the reaction was concentrated under reduced pressure affording crude imine as light brown solid (2.42 g, 98% yield) that was used directly in the next step.

The imine (2.3 g, 8.3 mmol) was dissolved in toluene (15 mL) and diethyl *H*-phosphonate [HP(O)(OEt)_2_] (1.07 mL, 8.3 mmol) was added in one portion. The resulting reaction mixture was heated at 120 °C for 8 h and then cooled down to room temperature and concentrated under reduced pressure. The resulting brown thick oil was essentially pure aminophosphonate ester (3.28 g, 95% yield) and was used directly in the next step.

The crude aminophosphonate ester (3.0 g, 7.22 mmol) was dissolved in toluene (30 mL) and aq. 6 M HCl (10 mL) was added. The resulting biphasic reaction mixture was vigorously stirred and heated at 120 °C for 8 h. After that time, the reaction was cooled down to room temperature, filtered off under vacuum over a glass sinter and the filtrate was transferred to a separatory funnel where layers were separated. The organic layer was discarded and the aqueous phase was evaporated to dryness under reduced pressure affording crude 1-amino-1-(3-thienyl)methylphosphonic acid hydrochloride as viscous oil. The crude product was dissolved in methanol (20 mL) and propylene oxide (20 mL) was added dropwise. The resulting mixture was left to stand at room temperature for 1 h and then pb laced in the freezer until next day. After that time the formed 1-amino-1-(3-thienyl)methylphosphonic acid was filtered off under vacuum over a glass sinter, washed with methanol (2 × 10mL) and dried on air (white non-hygroscopic powder, 0.84 g, yield 60%). MS(TOF MS ES+) Calcd for C_5_H_8_NO_3_PS [M + H]^+^ 194.0041; found: 194.0041. The assignment of signals on ^1^H and ^13^C NMR has been confirmed by ^1^-^1^H COSY, ^1^H-^13^C HMQC and ^1^H-^13^C HMBC spectra.

^31^P NMR δ(ppm): 11.62; ^1^H NMR: δ(ppm): 4.71 (d, *J* = 15.9 Hz, 1 H, PC**H**), 7.21 (d, *J* = 4.7 Hz, 1 H, 4-tiophene), 7.45–7.58 (m, 2 H, 2-tiophene, 5-tiophene); ^13^C NMR δ(ppm): 51.53 (d, *J* = 146.5 Hz, 1 C, P**C**), 128.22 (d, *J* = 7.7 Hz, 1 C, 2-tiophene), 129.61 (d, *J* = 3.7 Hz, 1 C, 4-tiophene), 130.41 (d, *J* = 1.0 Hz, 1 C, 5-tiophene), 134.45 (d, *J* = 4.9 Hz, 1 C, 3-tiophene).

### Synthesis of 1-amino-1-(4-imidazole)methylphosphonic acid (**2**)

Neat benzhydrylamine (1.53 mL, 8.9 mmol) was injected at room temperature to the solution of 4-imidazolecarboxaldehyde (1.0 g, 10.0 mmol) in EtOH (30 mL) and the reaction was stirred at 78 °C for 5 h. After that time the reaction was concentrated under reduced pressure affording crude imine as light-yellow solid (2.63 g, 97% yield) that was used directly in the next step.

The imine (2.5 g, 9.61 mmol) was dissolved in toluene (20 mL) and diethyl H-phosphonate [HP(O)(OEt)_2_] (1.24 mL, 9.61 mmol) was added in one portion. The resulting reaction mixture was heated at 120 °C for 8 h and then cooled down to room temperature and concentrated under reduced pressure. The resulting yellow solid was essentially pure aminophosphonate ester (3.64 g, 95% yield) and was used directly in the next step.

The crude aminophosphonate ester (3.5 g, 8.80 mmol) was dissolved in toluene (30 mL) and aq. 6 M HCl (15 mL) was added. The resulting biphasic reaction mixture was vigorously stirred and heated at 120 °C for 8 h. After that time, the reaction was cooled down to room temperature, filtered off under vacuum over a glass sinter and the filtrate was transferred to a separatory funnel where layers were separated. The organic layer was discarded and the aqueous phase was evaporated to dryness under reduced pressure affording crude 1-amino-1-(4-imidazole)methylphosphonic acid hydrochloride as viscous oil. The crude product was dissolved in methanol (30 mL) and propylene oxide (30 mL) was added dropwise. The resulting mixture was left to stand at room temperature for 1 h and then placed in the freezer until next day. After that time the formed 1-amino-1-(4-imidazole)methylphosphonic acid was filtered off under vacuum over a glass sinter, washed with methanol (2 × 10mL) and dried on air (white non-hygroscopic powder, 0.97 g, yield 63%). The spectroscopic characterization was in agreement with the data reported earlier in the literature [21]. MS(TOF MS ES+) Calcd for C_4_H_8_N_3_O_3_P [M + H]^+^ 178.0382; found: 178.0379. The assignment of signals on ^1^H and ^13^C NMR has been confirmed by ^1^-^1^H COSY, ^1^H-^13^C HMQC and ^1^H-^13^C HMBC spectra.

^31^P NMR δ(ppm): 7.53; ^1^H NMR: δ(ppm) 4.63 (d, *J* = 16.6 Hz, 1 H, PC**H**), 7.57 (s, 1 H, 5-imidazole), 8.68 (s, 1 H, 2-imidazole); ^13^C NMR δ(ppm): 46.43 (d, *J* = 138.3 Hz, 1 C, P**C**), 121.58 (d, *J* = 5.3 Hz, 1 C, 5-imidazole), 127.82 (d, *J* = 4.1 Hz, 1 C, 4-imidazole), 137.32 (1 C, 2-imidazole).

## Microorganism

*Rhodotorula mucilaginosa* (DSM 70,403) *Fusarium oxysporum* (DSM 12,646), *Trigonopsis variabilis* (DSM 70,714), *Beauveria bassiana* (DSM 875), *Cunninghamella elegans* (DSM 1908) and *Rhizopus oryzae* (DSM 1185), *Escherichia coli* (DSM 1116), *Bacillus subtilis* (DSM 10). were purchased from German Collection of Microorganisms and Cell Cultures (DSMZ, Germany). *Synechococcus bigranulatus* (CCALA 187) was purchased from the Culture Collection of Autotrophic Organisms (CCALA), Institute of Botany, Academy of Sciences, Czech Republic. *Penicillium commune* IAFB 2513 and *Aspergillus niger* IAFB 2301 were identified and deposited in Collection of Industrial Microorganisms (WDCM212) in Institute of Agricultural and Food Biotechnology (Poland). *Penicillium funiculosum* (Thom) S3 was isolated from soil sample and identified by DSMZ as reported previously [22]. Fungal strains were generous gift from following institutions: *Aspergillus parasiticus* (NRRLY 2999)—Andolu University (Turkey), *Mucor circinelloides*—Lodz University of Technology (Poland); *Penicillium citrinum* University of Opole (Poland).

Microorganisms were routinely maintained on dedicated media as follows: molds (*F. oxysporum, B. bassiana, C. elegans, R. oryzae, P. commune, A. niger, P. funiculosum, A. parasiticus, M. circinelloides, P. citrinum*) on potato dextrose agar (PDA) which provided profuse sporulation suitable for inoculum collection, yeasts (*R. mucilaginosa, T. variabilis*) on universal medium for yeast (YM), bacteria (*B. subtilis, E. coli*) on nutrient agar (NA) and cyanobacteria (*S. bigranulatus*) on BG-11 medium.

Commercially available PDA and NA media were used (VWR Chemicals) and prepared according to producer instruction. YM was prepared according to the recipe of DSMZ (www.dsmz.de, medium no. 186). BG-11 medium was prepared according to the recipe reported in the literature [23].

Mold fungi and yeasts were used as a biocatalyst in biotransformation procedures, while prokaryotic organisms served as model organisms for the Alamar Blue® Assay (2.6).

### Biocatalyst preparation—microorganisms cultivation

#### Cultivation of filamentous fungi

Biotransformation experiments with biocatalysts of molds origin were carried out with MEP (Malt Extract Peptone broth, 2.3.2) or PDB liquid medium either commercially available from VWR Chemicals and prepared as recommended or prepared according to the recipe on DSMZ website (medium no. 129). In latter case 200 g of potatoes were sliced and boiled in 1 L of water for 1 h. Then mixture was filtered, 20 g of glucose was added, and final volume adjusted up to 1 L with distilled water.

Spore suspensions were prepared with sterile 0.05% water solution of Triton X-100, quantified by vital count and stored at 4 °C. 500 µL of mentioned spore suspension (20,000 spores/µL) were used to inoculate Erlenmeyer flasks (250 ml) containing 100 ml of PDB or MEP depending on the experiment type. Cultures were grown on rotary shaker (130 rpm) at 24–26 °C until the late log phase. Mycelium was harvested by vacuum filtration onto filter paper and rinsed with distilled water. In the case of *F. oxysporum*, and *B. bassiana* cells were separated by centrifugation (20 °C, 5000 rpm, 10 min). Obtained biomass was used in biotransformation process.

#### Cultivation of yeast

Yeasts were cultivated using PDB medium (described above) or Malt Extract Peptone broth (MEP) or YM both prepared according to DSMZ instructions. Malt Extract Peptone broth (MEP) (DSMZ medium no. 90) is consisted of malt extract (30 g), soya peptone (3 g) and distilled water (1 L). Universal medium for yeasts (YM) (DSMZ medium no. 186) contains yeast extract (3 g), malt extract (3 g), peptone from soybeans (5 g), glucose (10 g) and distilled water (1 L). 1 mL portions of 3-day liquid culture were used to inoculate Erlenmeyer flasks (250 ml) containing 100 ml of medium and incubated at 24–26 °C on rotary shaker (130 rpm) until the end of logarithmic phase of growth. Yeast biomass was separated by centrifugation (20 °C, 5000 rpm, 10 min) and used for biotransformation processes.

#### Cultivation of bacteria and cyanobacteria

Bacteria (*E. coli, B. subtilis*) were cultivated on standard, commercially available LB medium (VWR Chemicals). 1-day liquid culture were used to inoculate Erlenmeyer flasks (250 ml) containing 100 ml of LB medium and incubated at 37 °C on rotary shaker (130 rpm).

*S. bigranulatus* were cultivated in Erlenmeyer flasks containing 100 mL of BG-11 medium [23]. Cyanobacteria were cultivated 21 days under continuous illumination at 7–12 µmol photons m^− 2^ s^− 1^ (Power Glo fluorescent bulb, 8 W, Hagen) at 28 °C under stationary conditions.

#### Biotransformation procedure

Method A1: Biomass received after cultivation (5 g) was suspended in 50 mL of distilled water containing 3 mM of substrate (29.0 mg of substrate **1** or 26.5 mg of substrate **2**). Bioconversions were carried out for 24–72 h (Tables 2, 3 and 4) at room temperature with shaking (135 rpm).

Method A2: Bioconversion was carried out according to Method A1 with the additional biocatalyst starvation period—the biomass was preincubated for 24–48 h. under deficiency of the nutrients (Tables 2, 3 and 4). After this time, 3 mM of substrate **1** or **2** was added and then bioconversion was carried out for the 1–3 days- depending on the experiment.

After biotransformation, biomass was removed by filtration or centrifugation, and supernatants were evaporated under reduced pressure using rotary evaporator. The resulting precipitates were analyzed by NMR spectroscopy.

#### Enantiomeric excess assignment

Optical purity of obtained product was evaluated with the ^31^P NMR spectroscopy recorded with addition of α-cyclodextrin applied as the chiral solvating agent (CSA). Enantio-discrimination efficiency depends on the pH values of the samples, because it influences the formation of the guest–host complexes between cyclodextrin and chiral compound [24–28]. So Table 5 includes data about the optimal pH (pD) values for particular NMR analysis.

**Table 5:** The dependence of ^31^P NMR chemical shifts differences (Δδ) on the pD values, recorded in the presence of α-cyclodextrin (100 mM) for racemic mixture of compound **1** and **2** (10 mM)

pD	2.5	4.8	7.5	10.3	11.0	12.0
Compound 1	0.04	0.01	0.00	0.00	0.18	0.24
**pD**	**2.4**	**4.0**	**7.1**	**10.0**	**11.0**	**12.0**
Compound 2	0.11	0.00	0.01	0.07	0.11	0.09

As it is shown above (Table 5) the biggest shifts difference (Δδ) for enantiomers of tested substrates **1** and **2** was set as pD 11–12 and such parameters were applied for further samples preparation. ^31^P NMR spectrum of racemic mixture of compound **1** with addition of α-cyclodextrin is presented on Fig. 2c, while the spectrum of compound **2** is shown on Fig. 3b.

^31^P NMR samples were prepared as follows: after biomass separation, the supernatant was evaporated, and finally product was dissolved in deuterium oxide (600 µL). Then α-cyclodextrin (100 mM) was added to the sample as a chiral solvating agent. The pH value of samples was adjusted to the pH ≈ 11–12 with NaOD solution and analyzed.

#### Absolute configuration assignment

After biotransformation, products were purified using Medium-Pressure Liquid Chromatography system (MPLC): Combi Flash® Rf 150 and reversed phase column PuriFlash C18-HP,15 μm, 120 g and then High Pressure Preparative Liquid Chromatography System (Teledyne ISCO ACCQPrep HP125) with reversed phase column RediSep Prep C18, 5 μm, 250 mm.

General procedure of purification using MPLC: 50 min of isocratic flow of pure water, 20 min from 0 to 100% of acetonitrile in water, 10 min of isocratic flow of pure acetonitrile; flow 10 mL/min, death time 3 min, R_f1 _= 7 min (compound **1**), R_f2 _= 8 min (compound **2**).

General procedure of purification using HPLC: 35 min of isocratic flow of pure water, 10 min from 0 to 100% of acetonitrile in water, 10 min of isocratic flow of pure acetonitrile; flow 10 mL/min, death time 7 min, R_f1 _= 9.5 min (compound 1), R_f2 _= 10 min (compound **2**).

Product was dissolved in 1 N NaOH and then optical rotation was measured. The optical rotation values were as follows: for 1-amino-1-(3-thienyl)methylphosphonic acid (**1**) (*e.e.* 55%; c = 1.27) ${\left[\alpha \right]}_{D}^{22}$= − 7.9 and for 1-amino-1-(4-imidazole)methylphosphonic acid (**2**) (*e.e.* 30%; c = 10) ${\left[\alpha \right]}_{D}^{22}$= − 1.3.

Absolute configuration of the 1-amino-1-(3-thienyl)methylphosphonic acid (**1**) was established using Mosher’s method and was compared to literature data for similar compound: 1-amino-1-(2-thienyl)methylphosphonic acid [29].

2-hydroxy-2-(ethoxyphenylphosphinyl)acetic acid (1.59 mmol, 373 mg) was mixed with *N,N,N’,N’*-tetramethyl-*O*-(1*H*-benzotriazol-1-yl)uronium hexafluorophosphate (1.91 mmol, 870 mg, HBTU), 1-amino-1-(3-thienyl)methylphosphonic acid (**1**) (2.07 mmol, 400 mg) and trimethylamine (3.19 mmol, 0.445 mL). This mixture was solved in acetonitrile (10 mL) and stirred for 72 h at room temperature. After that time, ethyl acetate (10 mL) was added to the resulting solution and the mixture was washed with 10 mL of 3 M HCl. Organic phase was dried with the anhydrous magnesium sulfate, then filtered and the liquid residues were evaporated. The obtained products were purified by medium pressure chromatography (MPLC) [15] and analyzed by MS: MS(TOF MS ES+) Calcd for C_15_H_16_F_3_NO_5_PSNa [M + Na]^+^ 432.0258; found: 432.0256.

1-(3-thienyl)-1-(3,3,3-trifluoro-2-methoxy-2-phenylpropylamido)-methylphosphonic acid **3**.

Isomer (*R*,*S*).

^31^P NMR δ(ppm): 18.22; ^1^H NMR: δ(ppm) 3.33 (s, 3 H, OC**H**_3_), 5.34–5.49 (m, 1 H, PC**H**), 6.86 (d, *J* = 5.8 Hz, 1 H, C_4_**H**_3_S, H-4), 6.94 (s, 1 H, C_4_**H**_3_S, H-2), 7.01 (m, 1 H, C_4_**H**_3_S, H-5), 7.23–7.56 (m, 5 H, C_6_**H**_5_), 7.74 (d, *J* = 9.1 Hz, 1 H, N**H**); ^13^C NMR δ(ppm): 47.92 (d, *J* = 153.1 Hz, 1 C, P**C**), 55.45 (1 C, O**C**H_3_), 84.35 (q, *J* = 25.7 Hz, 1 C, **C**(CF_3_)), 123.47 (1 C, **C**_4_H_3_S, C-2), 123.95 (q, *J* = 290.5, **C**F_3_), 126.13 (1 C, **C**_4_H_3_S, C-5), 127.52 (d, *J* = 3.4 Hz, 1 C, **C**_4_H_3_S, C-4), 127.68 (1 C, **C**_6_H_5_), 128.39 (1 C, **C**_6_H_5_), 128.80 (2 C, **C**_6_H_5_), 129.92 (1 C, **C**_6_H_5_), 132.57 (1 C, **C**_6_H_5_), 135.42 (1 C, **C**_4_H_3_S, C-3), 169.18 (1 C, **C**ON).

Isomer (*S*,*S*).

^31^P NMR δ(ppm): 18.31; ^1^H NMR: δ(ppm) 3.19 (s, 3 H, OC**H**_3_), 5.34–5.49 (m, 1 H, PC**H**), 7.02 (m, 1 H, C_4_**H**_3_S, H-4), 7.12 (m, 1 H, C_4_**H**_3_S, H-5), 7.17 (s, 1 H, C_4_**H**_3_S, H-2), 7.23–7.56 (m, 5 H, C_6_**H**_5_), 7.89 (d, *J* = 9.1 Hz, 1 H, N**H**); ^13^C NMR δ(ppm): 47.99 (d, *J* = 153.1 Hz, P**C**), 55.02 (1 C, O**C**H_3_), 84.41 (q, *J* = 25.7 Hz, 1 C, **C**(CF_3_)), 123.54 (1 C, **C**_4_H_3_S, C-2), 124.06 (q, *J* = 290.5, **C**F_3_), 126.38 (1 C, **C**_4_H_3_S, C-5), 127.44 (d, *J* = 3.4 Hz, 1 C, **C**_4_H_3_S, C-4), 127.68 (1 C, **C**_6_H_5_), 128.39 (1 C, **C**_6_H_5_), 129.02 (2 C, **C**_6_H_5_), 130.00 (1 C, **C**_6_H_5_), 131.74 (1 C, **C**_6_H_5_), 135.42 (1 C, **C**_4_H_3_S, C-3), 166.86 (1 C, **C**ON).

Absolute configuration of 1-amino-1-(4-imidazole)methylphosphonic acid **2** was established tentatively by optical rotation measurement, using the model—similar compound: 1-aminophenylmethanephosphonic acid. As a result: (*S*)-1-amino-1-(4-imidazole)methylphosphonic acid **2** was received, what was confirmed by the optical rotation measurement ${\left[\alpha \right]}_{D}^{22}$ = −1.3 (1 M NaOH, c = 10, *e.e. *= 30%)) [30].

#### Alamar Blue® assay

Alamar Blue® Assay allowed determining the antimicrobial activity of tested heterophosphonates. The 1-day liquid culture of microorganisms, (*E. coli*, *B. subtilis*) was prepared on standard LB medium. The absorbance of the liquid culture was adjusted to 0.125 optical density (OD) at 550 nm using a spectrophotometer. 3-day liquid culture of *R. mucilaginosa* was prepared on PDB medium, 21-day culture of *S. bigranulatus* on BG-11 medium and *A. niger* was cultivated 5 days on Potato Dextrose Agar (PDA). *R. mucilaginosa* and *S. bigranulatus* cells, as well as *A. niger* spores were counted on cytometer (BD FACSVerse™).

10 different concentrations (1024, 512, 256, 128, 64, 32, 16, 8, 4, and 2 µg/mL) of the compound **1** and **2** were prepared using two-fold serial dilution method with medium (LB Broth for bacteria, BG-11 for cyanobacteria and PDB for fungi). Aliquots were distributed as follows: 900 µL of tested compound solution prepared in liquid medium or sterile DI water (negative controls) and 100 µL of the liquid cultures were added to each Eppendorf tube (final cell concentration in tubes was 5·10^5^/mL). Experiments were done in triplicate for every set of data. The Eppendorf tubes were incubated for 24 h under the proper incubation temperature: either 26 or 37 °C for the fungi and bacteria, respectively. Tested compounds react with Alamar Blue® (Bio-Rad) and as a result resazurin is reduced to resorufin. After incubation was accomplished, biomasses were separated by centrifugation, and the pellets were rinsed with distilled water (3 times) to remove tested compounds. Then, biomass was suspended in 900 µL of distilled water and 100 µL of the reagent Alamar Blue® was added to each tube and absorbance was monitored at 570 nm and 600 nm (LAMBDA^TM^ XLS, PerkinElmer). If the obtained results exceeded the scale of the experiment (1024 µg/mL), the tests were repeated in a wider range of concentrations (10; 8; 5; 4; 2,5; 2; 1,25; 1 mg/mL).

## Data Availability

A significant part of the data generated and analysed in this study have been included in the article. Any further details about datasets used and analysed are available from the corresponding author on reasonable request.

## Abbreviations

CSA: Chiral solvating agent
MIC: Minimal inhibitory concentration
α-CD: α-Cyclodextrin
MPLC: Medium-pressure liquid chromatography system
MTPA: α-Methoxy-α-trifluoromethylphenylacetic acid
PDB: Potato dextrose broth
YM: Universal medium for yeasts
MEP: Malt extract peptone broth
PDA: Potato dextrose agar
DSMZ: German Collection of Microorganisms and Cell Cultures
